# Alpha-Melanocyte Stimulating Hormone (α-MSH) Is a Post-Caspase Suppressor of Apoptosis in RAW 264.7 Macrophages

**DOI:** 10.1371/journal.pone.0074488

**Published:** 2013-08-29

**Authors:** Andrew W. Taylor

**Affiliations:** Department of Ophthalmology, Boston University School of Medicine, Boston, Massachusetts, United States of America; University of Birmingham, United Kingdom

## Abstract

The neuropeptide alpha-melanocyte stimulating hormone (α-MSH) is an important regulator of immune cell activity within the immunosuppressive ocular microenvironment. Its constitutive presence not only suppresses macrophage inflammatory activity, it also participates in retinal pigment epithelial cell (RPE) mediated activation of macrophages to function similar to myeloid suppressor cells. In addition, α-MSH promotes survival of the alternatively activated macrophages where without α-MSH RPE induce apoptosis in the macrophages, which is seen as increased TUNEL stained cells. Since there is little know about α-MSH as an anti-apoptotic factor, the effects of α-MSH on caspase activity, mitochondrial membrane potential, Bcl2 to BAX expression, along with TUNEL staining, and Annexin V binding were examined in RAW 264.7 macrophages under serum-starved conditions that trigger apoptosis. There was no effect of α-MSH on activated Caspase 9 and Caspase 3 while there was suppression of Caspase 8 activity. In addition, α-MSH did not improve mitochondrial membrane potential, change the ratio between Bcl-2 and BAX, nor reduce Annexin V binding. These results demonstrate that the diminution in TUNEL staining by α-MSH is through α-MSH mediating suppression of the apoptotic pathway that is post-Caspase 3, but before DNA fragmentation. Therefore, as α-MSH promotes the alternative activation of macrophages it also provides a survival signal, and the potential for the caspases to participate in non-apoptotic activities that can contribute to an immunosuppressive microenvironment.

## Introduction

The neuropeptide alpha-Melanocyte Stimulating Hormone (α-MSH) is a thirteen amino acid peptide derived from endopeptidase cleavage of proopiomelanocortin hormone produced by the hypothalamus, monocytes, and retinal pigment epithelial cells (RPE) [1–4]. It is a neuropeptide that has an important role in metabolic and immune homeostasis. The neuropeptide suppresses inflammation mediated by both innate and adaptive immune responses [2,5]. It suppresses NF-κB activation along with p38 MAPK phosphorylation [6–8]. The neuropeptide promotes the alternative activation of endotoxin-stimulated macrophages by inducing IL-10 and TGF-β production [4,9]. In addition, it suppresses antigen presenting cells (APC) from activating effector T cells while promoting the APC to activate antigen-specific Treg cells [10–12].

The neuropeptide α-MSH is a central mediator of immunosuppression within the healthy ocular microenvironment [13,14]. In the anterior segment of the eye, the constitutive presence of α-MSH along with other neuropeptides and soluble factors participates in aqueous humor suppression of inflammation. Moreover, α-MSH is responsible for aqueous humor induction of regulatory T cells [15]. In the retina, the production of α-MSH and Neuropeptide Y (NPY) by the healthy RPE monolayer promotes expression of myeloid suppressor cell-like characteristics, and tolerance-mediating activity in macrophages and microglial cells [16]. When the α-MSH is neutralized, the RPE promotes activation of inflammatory activity in macrophages, similar to M1 macrophages. In addition, there is an increase in TUNEL staining of these macrophages in culture. By adding back α-MSH, the soluble factors produced by wounded-RPE will mediate expression of myeloid suppressor cell-like characteristics in macrophages. Also, there is a significant reduction in TUNEL staining. While this demonstrates that α-MSH has an important role in RPE mediated modulation of macrophage and microglial cell functionality to promote and maintain immune privilege and a healthy ocular microenvironment, it also suggests that α-MSH protects macrophages from apoptotic signals.

There are a few reports of α-MSH promoting cell viability in astrocytes, hypothalamic neurons, melanocytes, and renal tubular cells under apoptotic conditions, but none on macrophages [17–20]. In addition, it is unclear whether α-MSH suppresses any signal associated with apoptosis, nor how α-MSH could affect the cascade of activity associated with the mechanisms of apoptosis. Therefore, using the macrophage cell line, RAW 264.7, that express multiple pathways of apoptosis when serum starved [21–23], we examined the potential for α-MSH to suppress the apoptotic pathway and promote cell viability.

## Methods

### Cells, Reagents, Antibodies

The RAW 264.7 (ATCC, Manassas, VA) macrophage cells were maintained in complete media of RPMI 1640 (Lonza Walkersville, Walkersville, MD) supplemented with 10 μg/ml gentamicin (Sigma Aldrich, St. Louis, MO), 0.01M Hepes, 1x NEAA mixture, 1mM Sodium pyruvate (Lonza Walkersville), and 10% fetal bovine serum (Lonza Walkersville). For serum free conditions the serum was omitted and replaced with a 1/500 dilution of ITS+ media supplement (Sigma Aldrich). This serum free media is what was used to study the effects of neuropeptides on immune cells within the ocular microenvironment to mimic the ocular tissue environment behind its blood barrier [16]. The neuropeptide α-MSH was purchased from Bachem (Torrance, CA) reconstituted in 0.01 M PBS pH = 7.0, aliquoted, and stored at -80°C and thawed once for use. The anti-Caspase 8 antibody that detects both the precursor, and the p18 activation fragment of Caspase 8, and the anti-Caspase 9 antibody that detects precursor and activation fragments of Caspase 9 were purchased from Santa Cruz Biotechnology (Santa Cruz, CA). The Caspase 3, 8 and 9 activity was detected using individual colorimetric kits (R&D Systems, Minneapolis, MN). Apoptosis was detected by flow cytometry using terminal deoxynucleotidyl transferase dUTP nick end labeling (TUNEL) Apo-Direct Flow Cytometry Kit (Chemicon (Millipore), Temecula, CA), and an Annexin V- FITC apoptosis detection kit (BioVision Inc, Milpitas, CA). For immunoblotting BAX and Bcl-2 the antibodies were purchased from Santa Cruz Biotechnology. A cell permeable cationic dye, Mito Flow (Cell Technologies, Mountain View, CA) was used to assay for mitochondrial membrane potential designed for flow cytometry analysis.

### Cell culturing, viability, and TUNEL and Annexin V assay

The macrophages were cultured in serum-free media at 3 x 10^5^ cells / well in a 24 well plate. The macrophages were treated with α-MSH at 1 ng/ml, 3 ng/ml, 10 ng/ml, or untreated (identified as SFM in the figures). The cells were incubated for 18 hours, collected, washed with 0.01M PBS. The cell viability was detected by standard trypan-blue execution staining using a Bio-Rad TC-10 automatic cell counter. To detect for apoptosis the cultured macrophages were processed though either a TUNEL assay or an Annexin V binding assay. The processed cells were analyzed by flow cytometry. The gates were set for positive and negative apoptotic FITC-signals using internal assay kit controls. The cells treated with α-MSH were analyzed and the relative intensity of positive TUNEL or Annexin V-binding were determined by the ratio of the mean fluorescent intensity between the treated cells to the untreated (SFM) cells.

### Caspase Assays

The macrophages were cultured at 3 x 10^5^ cells/well in a 24 well plate under serum free conditions, and treated with α-MSH. The cells were incubated for 18 hours, collected and lysed using the colorimetric buffers of the Caspase 3, Caspase 8, or Caspase 9 assay kits. In addition, the concentration of protein in the lysates was measured by Bio-Rad protein assay. The relative activity of the caspases to convert their respective substrates was measured by OD(405nm) per mg of protein lysate.

### Immunoblotting

The macrophages were cultured and treated as above in a 24 well plate. After 18 hours incubation the cells were washed once in PBS, and lysed with standard RIPA buffer supplemented with protease inhibitors. The lysate was applied to a 4-12% gradient Invitrogen NuPage gel with MOPS buffering, and reducing reagent. This was followed by protein transfer to nitrocellulose filter for immunoblotting. The filter was probed with anti-Caspase 8, anti-Caspase 9, anti-BAX, or anti-BCL-2 antibody followed by HRP-conjugated secondary antibody. The bands were visualized by chemoluminescence and imaged using a Bio-Rad Versa doc system. The image was further analyzed using Bio-Rad one system software to calculate the ratio of the band intensities between precursor and p18 activation fragment of Caspase 8, between Caspase 9 activation fragments to precursor protein. The band intensities of BAX and BCL-2 were normalized to beta-actin band intensity of the same stripped and anti-beta-actin reprobed membrane. The normalized values were used to calculate the ratio between the two proteins in the treated and untreated macrophages.

### Mitochondrial membrane integrity assay

The mitochondrial membrane potential was measured by incubating and treating the macrophages with α-MSH as was done above. In the last hour of incubation, mitochondrial membrane potential dye Mito-Flow was added to each cell culture. The cells were collect, assayed by flow cytometry, and the relative levels of mitochondrial retention of dye were determined. Dye retention, indicating intact mitochondrial membrane potential, appears as a high fluorescent intensity signal identified on the flow cytometry histograms.

### Phagocytic assay

Untreated macrophages were cultured under serum-free conditions for 24 hours, collected, and stained using the TUNEL assay as we had done before. These fixed and TUNEL stained cells were washed, and added at 1 x 10^5^ cells / culture of fresh macrophages treated with α-MSH as described above. One pooled preparation of TUNEL stained cells were used per experiment, with 70% of the cells staining for TUNEL as assayed by flow cytometry. After 18 hours of incubation, the floating cells and the scraped adherent cells were collected, washed, and assayed by flow cytometry for FITC staining, which indicated that the cells bound or took up the apoptotic cells or bodies with stained DNA fragments.

### Statistical Measurements

**Figure 1:**
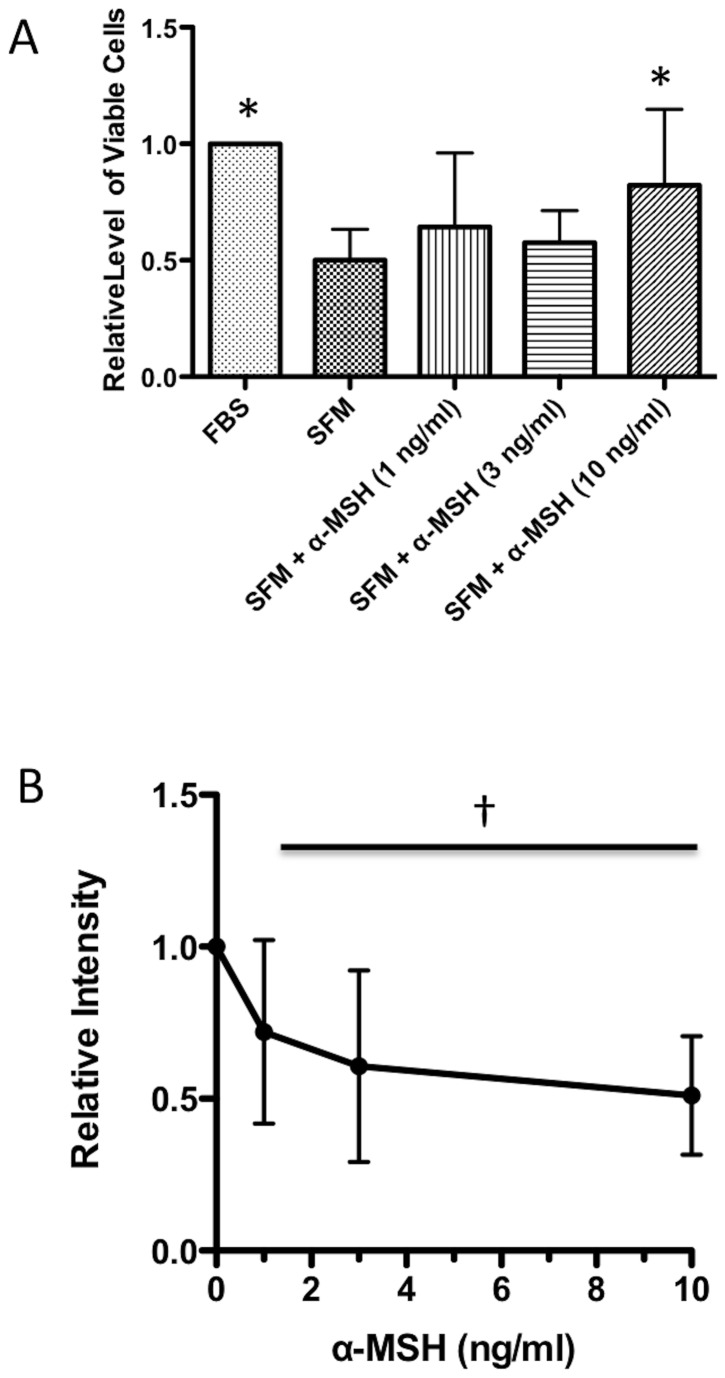
Effects of α-MSH on TUNEL staining of macrophages under serum-free conditions. The macrophages were incubated for 18 hours under serum-free conditions and treated with α-MSH at 1, 3, and 10 ng/ml. **A**. The cells were counted by trypan-blue exclusion. The mean relative levels of expected number of viable cells ± SD is presented. * Significance P <0.05 was detected in the cultures treated with 10 ng/ml compared to untreated cells (SFM) based on 8 independent experiments. **B**. The cells were stained using a TUNEL staining kit, and analyzed by flow cytometry. The mean fluorescent intensity of the TUNEL positive staining was normalized to the mean fluorescent intensity of TUNEL positive staining of the cells cultured under serum free conditions not treated with α-MSH (SFM). **†** There is a significant (P < 0.05 and Spearman coefficient r = -1) correlation with the decrease in TUNEL staining by increased concentrations of α-MSH treatment. The results are presented as the relative intensities ± SD of 3 independent experiments with the value of 1 for the SFM cells in each experiment.

All the statistical measurements presented in this manuscript used non-parametric Mann-Whitney t-test between treated samples and their respective controls. Non-parametric Spearman rank correlation analysis was used in Figure 1B. The threshold for statistical significance was defined as P ≤ 0.05. The results are presented as the mean ± standard deviations for the number of independent experiments stated in the figure legends. The flow cytometry histograms and digital photographs of immunoblots are representative of at least 3 independent experiments with similar results.

## Results

### The effects of α-MSH on TUNEL staining

The RAW 264.7 macrophage cells were treated with α-MSH and cultured under serum-free conditions for 18 hours. The concentrations of α-MSH tested (1, 3, and 10 ng/ml) were centered on the 3 ng/ml of α-MSH found in the conditioned media of healthy RPE eyecups incubated for 24 hours [16]. The cells were counted using trypan-blue exclusion, or TUNEL stained. The viable cell numbers of untreated macrophages decreased by 50%; however, in the cultures of macrophages treated with α-MSH at 10 ng/ml there was no change in viable cell numbers (Figure 1A). When the cells were TUNEL stained, there was a significant suppression in the relative intensity of TUNEL positive staining in cultures of macrophages treated with α-MSH (Figure 1B). These results suggest that as α-MSH promoted cell viability, it suppressed the level of DNA fragmentation in the macrophages.

### The effects of α-MSH on Caspase 9, Caspase 8, and Caspase 3 activity

**Figure 2:**
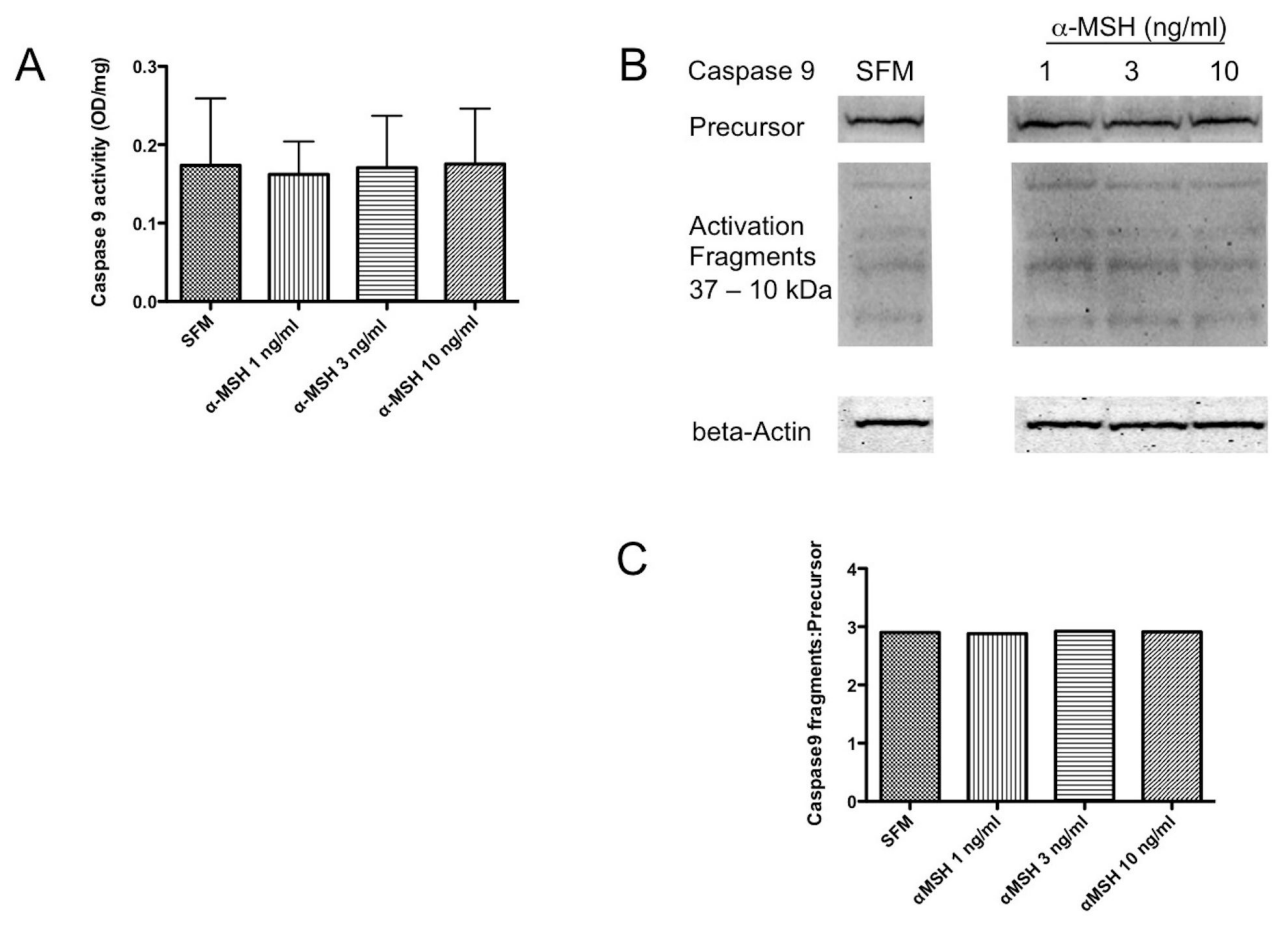
Effects of α-MSH treatment on Caspase 9 activity. The macrophages were cultured and treated as described in Figure 1 and the lysate was assayed for protein concentration, Caspase 9 activity, and immunoblotted for Caspase 9 protein. **A**. There was no significant difference seen between α-MSH treated and untreated (SFM) cells. Results presented are the mean ± standard deviation of 3 independent experiments. **B**. The lysates were immunoblotted with antibody that detected precursor and the activation fragments of Caspase 9. The band intensities were measured and made relative to beta-actin band intensity. **C**. The ratio of Caspase 9 activation fragments to precursor protein did not change between the untreated SFM cells and the α-MSH treated cells. These results are representative of 3 independent experiments showing that α-MSH has no effect on Caspase 9 activation.

**Figure 3:**
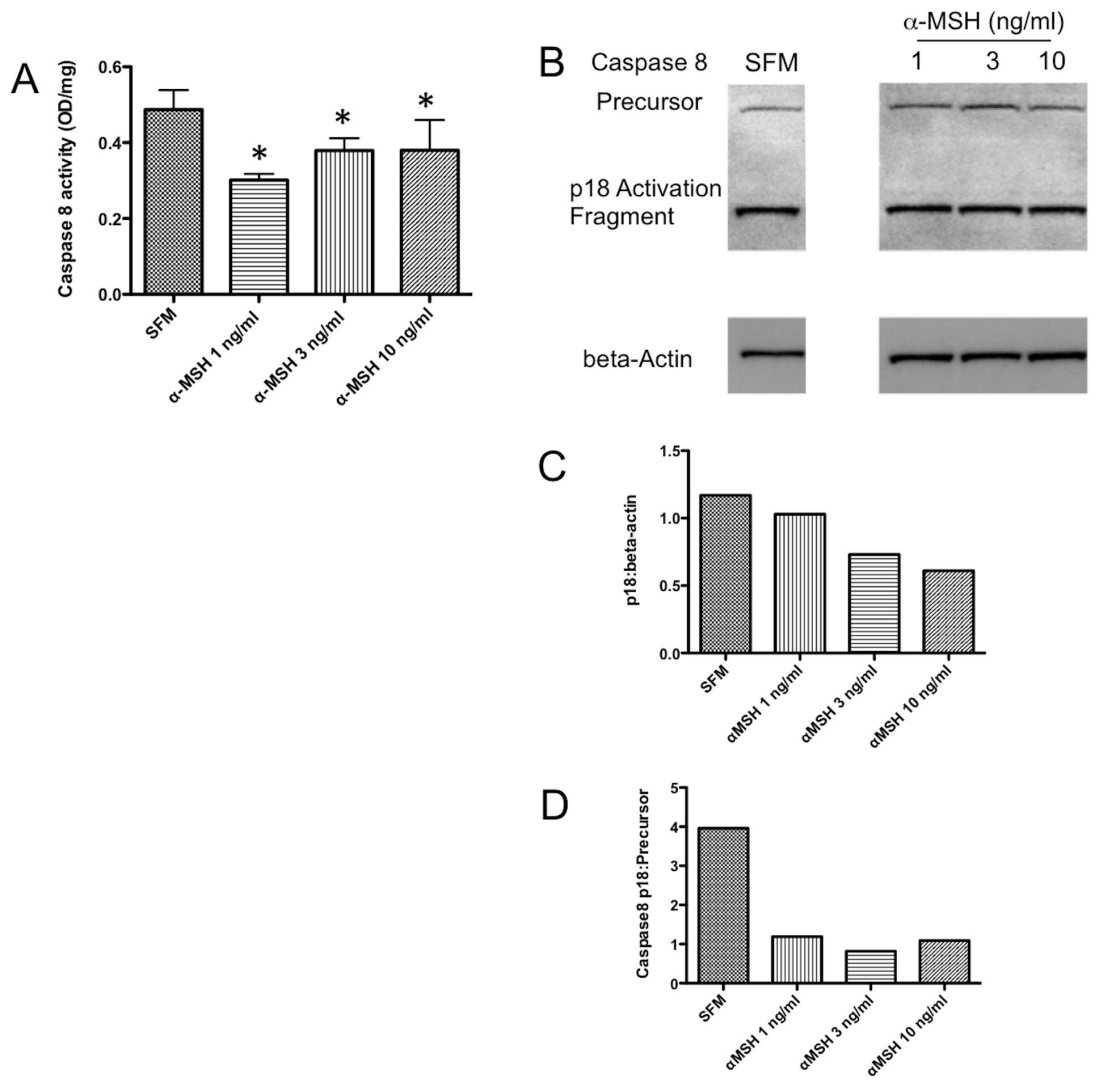
Effects of α-MSH treatment on Caspase 8 activity. The lysates were assayed for protein concentration, Caspase 8 activity, and immunoblotted for Caspase 8 protein. **A**. *Significant (P ≤ 0.05) differences in Caspase 8 activity were seen between untreated (SFM) cells and cells treated with 1 and 3 ng/ml of α-MSH. Results presented are the mean ± standard deviation of 4 independent experiments. **B**-**D**. The lysates were immunoblotted with antibody that detected precursor Caspase 8 and the p18 activation fragment. These results are representative of 3 independent experiments showing the relative expression of the p18 activation fragment band to beta-actin was less in the cells treated with α-MSH at 3 and 10 ng/ml and that the ratio of the activation fragment to precursor protein was diminished in all cells treated with α-MSH compared to the untreated cells (SFM). This shows that α-MSH treatment suppresses Caspase 8 activity.

To see if α-MSH had any effect on caspase activities within the macrophages under serum-free conditions, the α-MSH treated cells were lysed and assayed for Caspase 9 and Caspase 8 activity. The activity of Caspase 9 was detectible in the macrophages cultured without serum, and α-MSH had no effect on Caspase 9 activity (Figure 2A). In addition, immunoblotting for the precursor protein and activation fragments of Caspase 9 from cell lysates were unchanged with α-MSH treatment (Figure 2B). This suggests that intrinsic signals of apoptosis are most likely not suppressed by α-MSH treatment. In contrast there was significant suppression of Caspase 8 activity (Figure 3A). Also, α-MSH treatment reduced the expression of Caspase 8 p18 activation-fragments in the macrophages (Figure 3B, C). In addition, the ratio of Caspase 8 activation p18 fragments to Caspase 8 precursor proteins was suppressed by α-MSH at the concentrations tested (Figure 3D) These results demonstrate a suppression of Caspase 8 activity in the α-MSH treated macrophages mostly at lower concentration of α-MSH. It is possible that the changes in Caspase 8 activity with the α-MSH concentration is more related to increased cell numbers in the 10 ng/ml α-MSH treated cell cultures.

**Figure 4:**
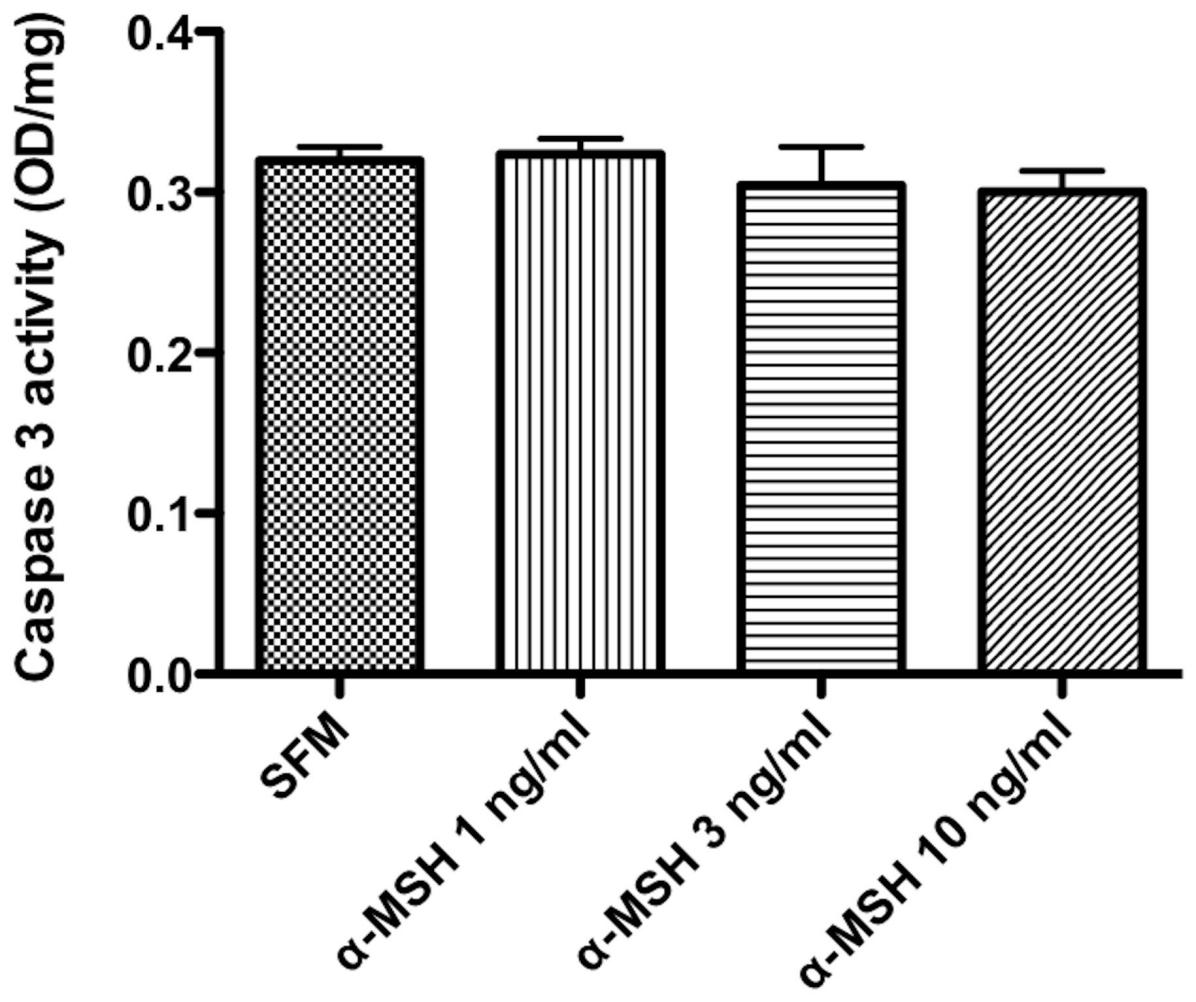
Effects of α-MSH treatment on Caspase 3 activity. The macrophages lysates were assayed for protein concentration and Caspase 3 activity. There was no significant change of Caspase 3 activity in cells treated with α-MSH compared to SFM cells. The graft is the mean ± SD of Caspase 3 activity (OD/mg total protein) of 3 independent experiments. The results show that α-MSH has no effect on Caspase 3 activity.

The cells were assayed for the activity of the final Caspase in the pathway, Caspase 3. The cells were treated with α-MSH as before, and when the lysates were assayed for Caspase 3 activity no change in activity was seen compared to the untreated cells (Figure 4). These findings demonstrate that the effects of α-MSH on caspase activation and activity are minimal to none suggesting the diminution of TUNEL staining, and the increase in cell viability mediated by α-MSH must be distal of Caspase 3 activation, but before DNA fragmentation.

### The effects of α-MSH on mitochondrial associated apoptotic activity

**Figure 5:**
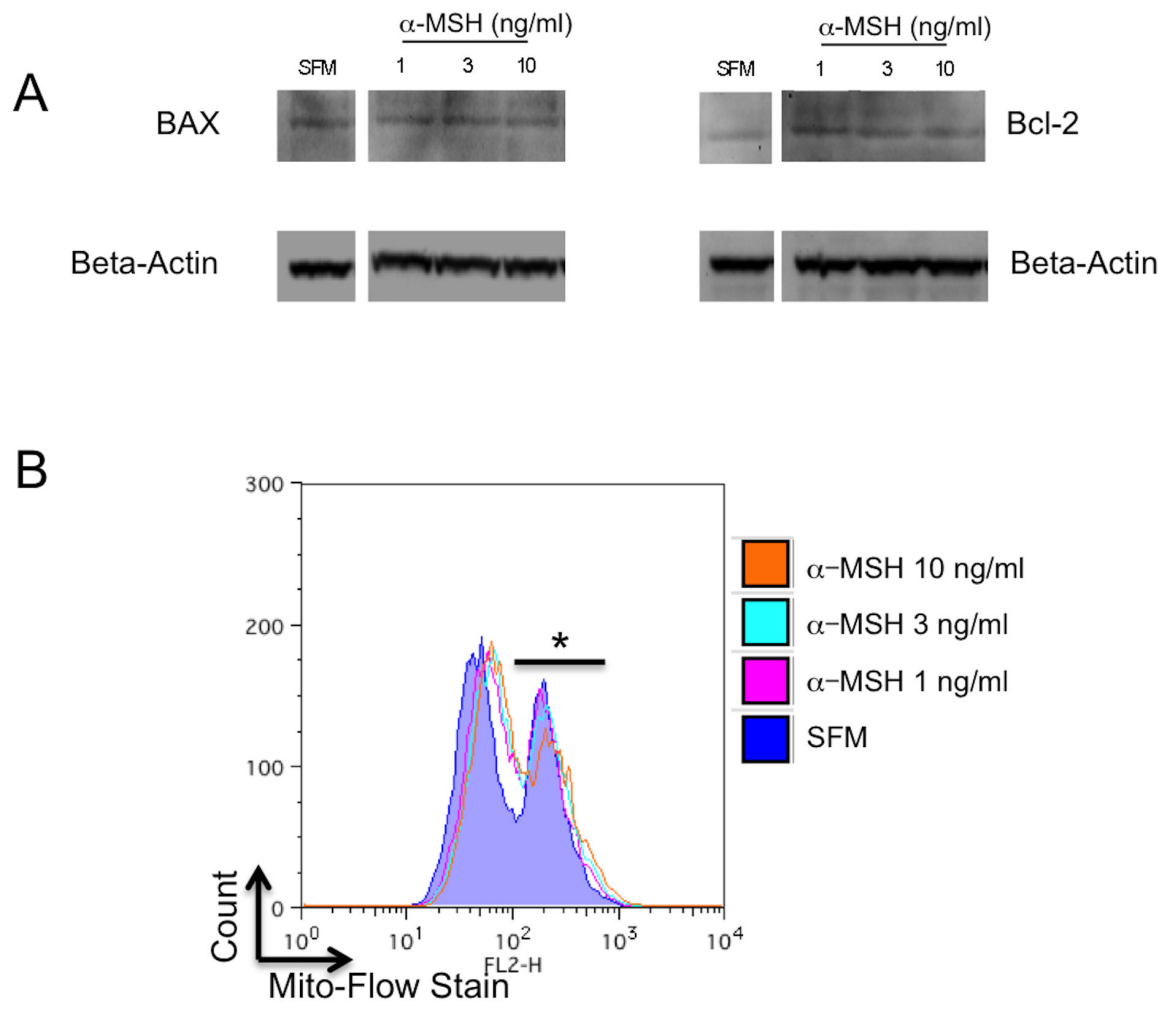
Effects of α-MSH on potential intrinsic mitochondrial pathways of apoptosis. **A**. The macrophages were cultured under serum free conditions and were lysed and immunoblotted for BAX and Bcl-2. There was no change in band intensities for BAX and Bcl-2 relative to beta-actin in α-MSH treated and untreated (SFM) cells, nor was there any change in the BAX to Bcl-2 ratio. These results are representative of 3 independent experiments. **B**. The macrophages were cultured as before, and in the last hour of incubation they were treated with Mito-Flow dye. The cells were assayed by flow cytometry for expression of retained mitochondrial dye in treated and untreated (SFM) macrophages. In all the cultures less than half the cells retained high levels of the dye (*) indicating mitochondrial membrane integrity, with greater than half the cells with low levels of dye indicating loss in mitochondrial membrane integrity. Treating the cells with α-MSH had no effect on this pattern of dye retention. The flow cytometry histogram presented is representative of 3 experiments with the same result of no measurable change seen related to α-MSH treatment. There is no effect on potential intrinsic apoptotic activity by α-MSH.

There is a possibility that α-MSH protects the cells from the stress of serum-free induced death by reducing mitochondrial stress. The cells were treated as before and lysed. The lysates were assayed for BAX and Bcl-2 by immunoblotting. There was no observable or measurable change in the expression of BAX or Bcl-2 or the ratio of the two with α-MSH treatment (Figure 5A). Mitochondrial stress was further assayed by loading the cells with Mito-Flow stain to assay for the integrity of the mitochondrial membrane potential. The cells were incubated under serum-free conditions, treated with α-MSH, and assayed by flow cytometry. There was no change in dye retention by the mitochondria in macrophages treated with α-MSH (Figure 5B). The results show that there was a loss in mitochondrial membrane potential in more than 50% of the macrophages under serum-free conditions treated or not treated with α-MSH. Therefore, α-MSH is not providing survival signals through changes in mitochondrial activity; moreover, it may allow for apoptotic signals to propagate from the stressed mitochondria.

### The effects of α-MSH on apoptotic body uptake

**Figure 6:**
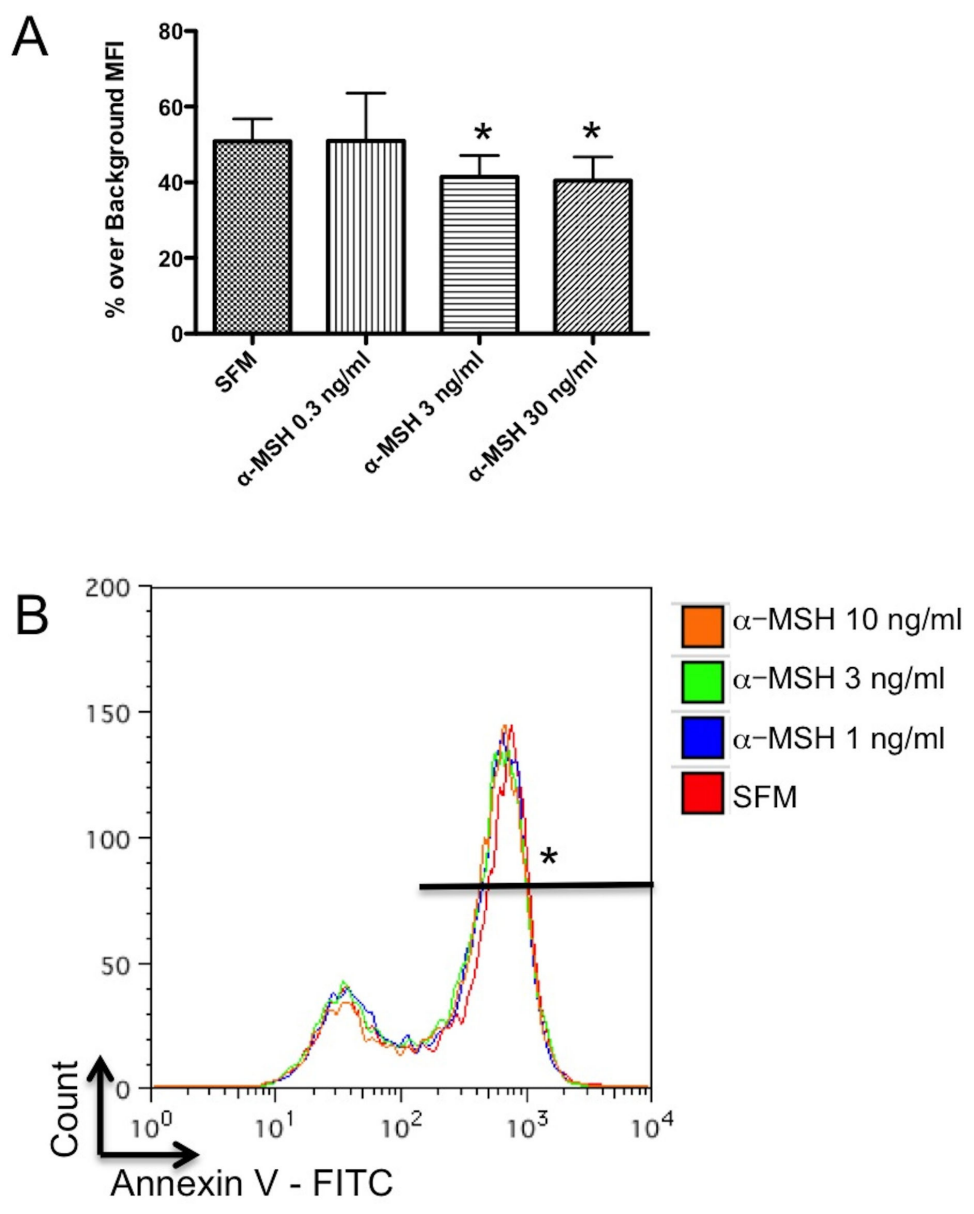
Effects of α-MSH on apoptotic body uptake and Annexin V binding. **A**. Untreated macrophages were cultured under the same conditions used for the TUNEL assay in Figure 1. These fixed and TUNEL stained cells were fed to fresh cultures of macrophages treated with α-MSH. The macrophages were assayed 18 hours by flow cytometry for FITC-staining. The mean percent ± SD of cells positive for FITC-staining over background of 4 experiments are presented with significant (P ≤ 0.05) suppression in cells treated with 3 and 10 ng/ml of α-MSH. These results suggest that there is a minor contribution to α-MSH diminution of TUNEL staining associated with α-MSH suppressing the take-up of DNA-fragment containing apoptotic bodies. **B**. The macrophages were cultured under serum free conditions and assayed by flow cytometry for Annexin V binding. No difference in Annexin V binding was seen between the untreated (SFM) and the α-MSH treated macrophages. The flow cytometry histogram presented is representative of 4 experiments with the same result of no measurable change seen related to α-MSH treatment. These results demonstrate that α-MSH does not suppress the initial steps in the apoptosis pathway.

Since the RAW cells are phagocytes, there is a possibility that the diminution of TUNEL staining in the α-MSH treated cultures is due reduced uptake of apoptotic bodies with TUNEL detectible DNA fragments. The cells were incubated under serum free conditions, fixed and TUNEL stained. These fixed and stained cells were added to new α-MSH-treated macrophage cultures under serum-free conditions. After incubation the macrophages were washed, assayed by flow cytometry. The analysis gated on intact cells, and the intensity of the TUNEL stain from bound or phagocytized material was measured. There was a significant suppression in the intensity of detectible TUNEL staining in the cells treated with 3 and 10 ng/ml of α-MSH (Figure 6A). This suggests that some of the suppressed TUNEL staining is due to α-MSH inhibiting the phagocytic uptake of apoptotic cells, which would be detected as a reduction in TUNEL staining.

### The effects of α-MSH on Annexin V binding of macrophages under serum-free conditions

The binding of Annexin V is a very early event in apoptosis. If α-MSH is suppressing the distal stages of apoptosis then Annexin V binding should not change. The cells were cultured as before, stained with Annexin V - FITC, and analyzed by flow cytometry. There was no change in Annexin V binding between the untreated and the α-MSH treated cells. Therefore, the effects of α-MSH on TUNEL staining and cell viability must be distal to the initial stimulation of the apoptotic pathway.

## Discussion

Our results demonstrated that while α-MSH caused a diminution of TUNEL staining in serum-starved macrophages, α-MSH had no effect on caspase 9 and 3 associated steps of the apoptotic pathway. This indicates that the effects of α-MSH are proximal to DNA fragmentation, but distal to Caspase 3 activity. In addition, there is a minor contribution to the diminution of TUNEL staining associated with α-MSH suppression of apoptotic body uptake by the phagocytic macrophages. There was α-MSH suppression of Caspase 8 activation, suggesting that α-MSH maybe limited to blocking the initial steps of the extrinsic apoptotic pathway, but not the intrinsic pathway of apoptosis mediated by Caspase 9. The results clearly show that α-MSH has no effect on Caspase 9 and mitochondrial activity (Bcl-2/BAX and membrane potential integrity) of the intrinsic pathway of apoptosis. Our results are similar to the findings of α-MSH protection of melanocytes from UVB-induced apoptosis where there is no effect of α-MSH on the apoptotic pathway, but there are increases in nucleotide excision repair activity to decrease UVB-induced DNA damage [17]. Therefore, the effects of α-MSH on TUNEL staining and cell viability in the macrophages must be post-caspase activity in the apoptotic pathway.

It has been shown that inhibiting phosphorylation of p38 MAPK inhibits the apoptosis pathway before DNA fragmentation [24–27]. It is not clear if p38 MAPK activation is directly involved in the activation of DNA fragmentation, or that its activation is a bystander indicator of Caspase 3 activation of MAP kinase kinases that initiates the process of DNA fragmentation. While the intracellular pathways of α-MSH mediated signaling are not fully understood, it is known that α-MSH suppresses the activation of both NF-kB and p38 MAPK through its melanocortin receptors MC1r and MC3r [6–8]. This suppresses inflammatory activity in the macrophages stimulated through Toll-like receptors (TLR) and pro-inflammatory cytokines. The suppression of NF-kB is α-MSH promoting IRAK-M binding to IRAK-1 to inhibit TLR signaling [28]. The suppression of p38 MAPK is though α-MSH activation of cAMP-dependent PKA [7,8]. Therefore, since the results show no effect of α-MSH on the initial steps and the caspase steps of apoptosis, and that α-MSH is known to suppress p38 MAPK phosphorylation, it suggests that the effects of α-MSH are on the post-caspase steps of apoptosis to promote cell survival.

The survival of such affected macrophages may be part of immune-homeostasis mediated by α-MSH within localized tissue microenvironments. We know that macrophages treated with α-MSH are suppressed in inflammatory activity, and may promote regulatory or tolerogenic immunity [4,9–12]. In the retina, α-MSH is part of RPE mediated induction of suppressor macrophages and similar characteristics in the retinal microglial cells [16]. However, the signaling pathways that result in activation of genes and functions of inflammation within macrophages are also signals of survival and resistance to apoptosis. Therefore, in the healthy retina α-MSH must be providing an anti-inflammatory signal, and a necessary survival signal for the immunosuppressive microglial to function and survive in the retina. Moreover, α-MSH may be part of a selective mechanism mediated by the RPE to exclude macrophages that are unresponsive to α-MSH immunosuppressive activity. In this way macrophages and possibly dendritic cells migrating into the retina would encounter the soluble apoptotic signal from RPE and will have to respond to α-MSH to survive, but by doing so makes them immunosuppressive. This selective process could explain why in the healthy retina the microglial cells are found to be characteristically uniform as myeloid suppressor cell-like [16].

Some of the caspases also have non-apoptotic activities [29]. The diversion of the caspases to other non-apoptotic activities could be the effects of α-MSH with saving the macrophages from apoptosis. A non-apoptotic activity of Caspase 8 is to activate NF-κB [30]. The suppression of Caspase 8 that was detected could be α-MSH suppressing another pathway to NF-κB activation. Maintaining non-apoptotic activities of Caspase 3 has benefits to ocular immunobiology. The non-apoptotic functions of Caspase 3 include suppression of MHC class II antigen expression and dendritic cell maturation [31]. This would greatly contribute to the immune privileged and immunosuppressive microenvironment of the retina. Since α-MSH can have a role in immune-homeostasis to resolve inflammation, it could mediate more than immunosuppression by maintaining in surviving cells caspase functionalities that contribute to resolving inflammation and promoting wound repair.

The neuropeptide α-MSH is not just an anti-inflammatory factor. It modulates the functionality of immune cells. This is seen in it promoting alternative activation of macrophages and dendritic cells in that they suppress inflammation and activate regulatory activity in T cells [4,9–12,32]. With NPY, α-MSH promotes expression of myeloid suppressor cell-like characteristics and activities in macrophages and microglial cells [16]. Along with these immunomodulating actions, α-MSH promotes survival at the later steps of apoptosis with the potential to further promote immunoregulation and immunosuppression by macrophages.
